# Characterization of the complete mitochondrial genome of *Caryopemon giganteus* Pic (Coleoptera: Chrysomelidae: Bruchinae)

**DOI:** 10.1080/23802359.2020.1719927

**Published:** 2020-01-29

**Authors:** Mingsong Wu, Xin Qian, Ming Qin, Tieyao Tu, Runzhi Zhang

**Affiliations:** a Key Laboratory of Plant Resources Conservation and Sustainable Utilization, South China Botanical Garden, Chinese Academy of Sciences, Guangzhou, Guangdong, China;; b College of Life Sciences, University of Chinese Academy of Sciences, Beijing, China;; c Key Laboratory of Zoological Systematics and Evolution, Institute of Zoology, Chinese Academy of Sciences, Beijing, China

**Keywords:** Bruchinae, Leguminosae, *Mucuna*, phylogeny, seed beetle

## Abstract

We sequenced, assembled, and annotated the complete mitochondrial genome of the seed beetle *Caryopemon giganteus*, which represents the first report in the tribe *Caryopemini* from the subfamily Bruchinae of Chrysomelidae. The circular mitochondrial genome of the species contains 15,727 bases, 13 protein-coding genes (PCGs), 22 tRNA genes, 2 rRNA genes, and a non-coding region. The GC content of the genome is 25.3%, which is higher than any other reported mitochondrial genomes within Bruchinae. The 16S ribosomal RNA gene and the 12S ribosomal RNA gene are 1284 and 835 bp in length, respectively. 12 PCGs started with the typical ATN codon, except for ND1 initiated with TTG. Five PCGs have the typical stop codon of TAA or TGA, while the remainder PCGs are terminated with incomplete stop codons (TA or T). The phylogenetic analysis based on a combination of 13 genes of the mitochondrial genomes of six species of Bruchinae and 23 species from other 10 subfamilies of Chrysomelidae recovered a generally well resolved and strongly supported tree topology, which shows that *C. giganteus* has the basalmost position in Bruchinae.

Bruchid beetles (Coleoptera: Chrysomelidae: Bruchinae) have the habit of feeding within seeds as larvae, so that they have the common name seed-beetles. More than 1700 seed-beetle species from 58 genera have been described (Johnson et al. 2004; Alvarez et al. 2005), of which 84% feed on seeds of the family Leguminosae (Johnson 1970). Seed-beetles were traditionally considered as a separate family Bruchidae within Chrysomeloidea (Crowson 1955; Duckett 1997; Verma 1998). Many researchers lowered seed-beetles to subfamily level based on molecular evidence (Bocak et al. 2014; Farrell and Sequeira 2004; Haddad and Mckenna 2016), with the subfamily Sagrinae placing as sister group within the family Chrysomelidae (Duckett et al. 2004; Farrell and Sequeira 2004). *Caryopemon giganteus* Pic belongs to subfamily Bruchinae of family Chrysomelidae (Lingafelter and Pakaluk 1997; Schmitt 1998; Verma and Saxena 1996), generally feeding on the seed of the genus *Mucuna* (Johnson 1981), and has a wide geographic distributional range from India to China (Li et al. 2016).

The materials were reared from the seeds of *Mucuna birdwoodiana* Tutch. collected from Dinghu mountain, Guangdong Province, China (N23°10′31.38′′, E112°32′25.64′′) in December 2018. The adults were stored in 100% ethanol at −20 °C before DNA extraction. The specimens and tissue samples were deposited in the Institute of Zoology (IOZ), Chinese Academy of Sciences. Total genomic DNA was extracted using the TIANamp Genomic DNA Kit (Tiangen Biotech Co., Ltd., Beijing, China). Next-generation sequencing was completed on the Illumina HiSeq platform and 150 bp paired-end reads were generated with insert size around 350 bp by Novogene (Beijing, China). In total, 4.69 GB of clean data were obtained for assembling the complete mitochondrial genome using the GetOrganelle pipeline (Jin et al. 2018). The complete mitochondrial genome sequences of *Acanthoscelides obtectus* Say (MF925724) and *Callosobruchus maculatus* Fab. (KY942060) were used as references to annotate the mitochondrial genome using the GeSeq (Tillich et al. 2017). Geneious prime 2019.2.3 (Biomatters Ltd., Auckland, New Zealand) was used to confirm the accuracy of the assembly with the Map to Reference tool, and to adjust the start/stop codons and intron/exon boundaries of the annotation. The annotated genome was deposited in GenBank under the accession number MN881034.

The complete mitochondrial genome of *C. giganteus* Pic is a double-stranded circular molecule of 15,727 bp in length, consisting of 13 protein-coding genes (PCGs), 22 tRNA genes, 2 rRNA genes, and a non-coding region. The size of the 16S ribosomal RNA gene is 1284 bp and the 12S ribosomal RNA gene is 835 bp. The composition of each base was calculated as A (38.4%), T (36.3%), C (14.8%), G (10.5%), with a GC content of 25.3%, which is higher than any other known genomes of Bruchinae (Yao et al. 2017; Song et al. 2018; Sayadi et al. 2017). Twelve PCGs started with typical ATN codon (ATC for *ND2*, *ATP8*,and *ND3*; ATT for *COX1*, *COX2*, *ND6*, and *ND5*; ATG for *ATP6*, *COX3*, *ND4*, *ND4L*, and *CYTB*), except for ND1 initiated with TTG. Two PCGs (*COX1* and *ND1*) terminated with TAG, three PCGs (*ND2*, *ATP6*, and *COX3*) terminated with TAA, while eight PCGs terminated with incomplete stop codons (TA or T).

To reveal the phylogenetic position of *C. giganteus* within the family Chrysomelidae, we downloaded 32 complete mitochondrial genomes from the GenBank, which contains 29 species from 11 subfamilies of Chrysomelidae. Three species of Lamiinae were used as outgroups. The phylogeny (Figure 1) of Chrysomelidae was reconstructed based on the concatenated matrix of amino acid sequences of 13 mitochondrial PCGs. MAFFT v.7.308 (Katoh and Standley 2013) was used to align the data matrix with default parameters. The maximum-likelihood (ML) phylogenetic tree was constructed using the RAxML-HPC 8.2.10 (Stamatakis 2014) on CIPRES cluster (https://www.phylo.org/), under the GTR + G substitution model as suggested by ModelFinder (Kalyaanamoorthy et al. 2017). Our phylogenetic analysis recovered a generally well resolved and strongly supported tree topology, which shows that *C. giganteus* has the basalmost position in Bruchinae. Our results could provide essential data to investigate the systematics and host-plant association patterns of seed-beetles in the future.

**Figure 1.**
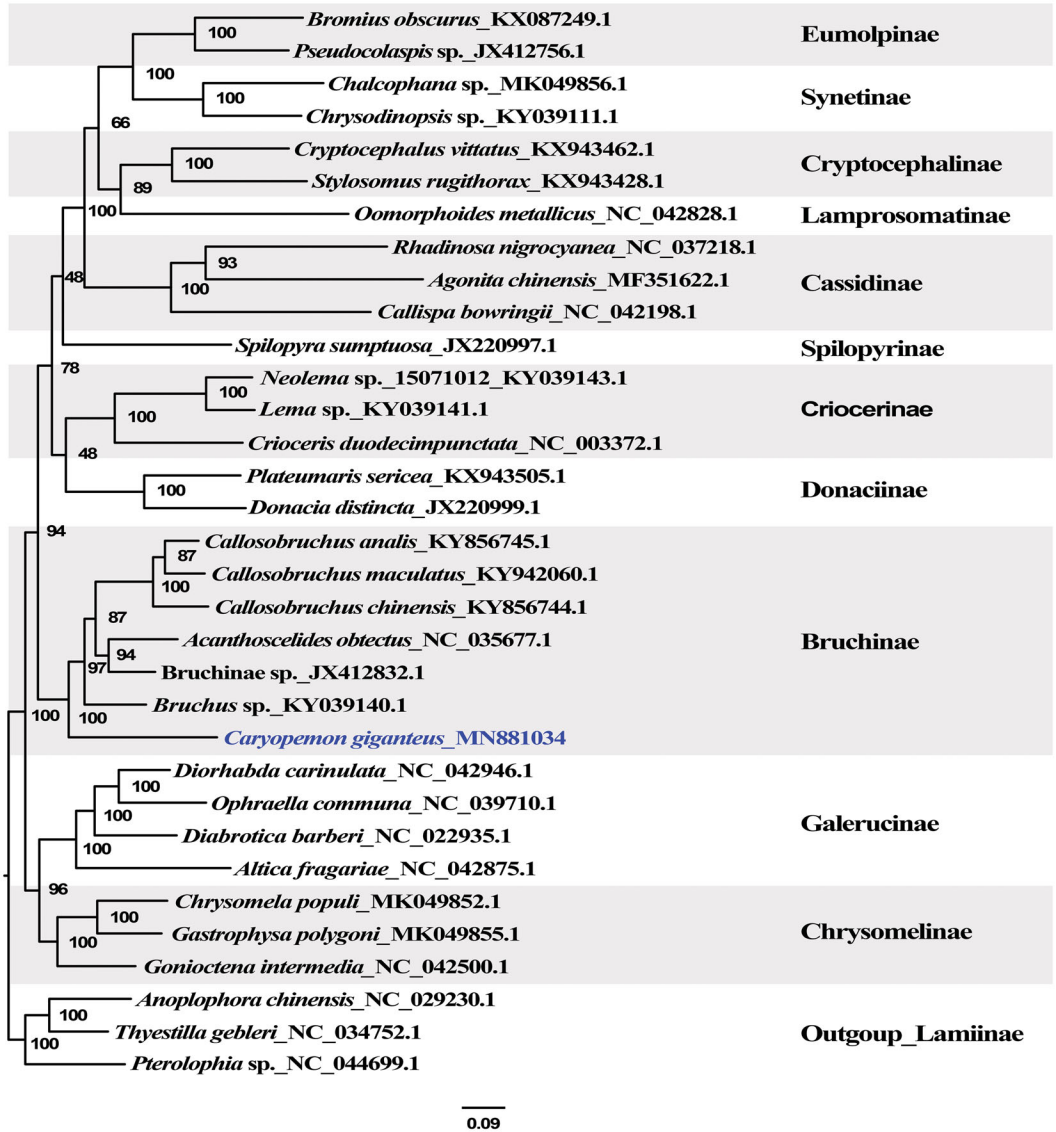
The maximum-likelihood (ML) phylogenetic tree based on the concatenated matrix of amino acid sequences of 13 mitochondrial PCGs. The numbers near the branch node are bootstrap support values.

## References

[CIT0001] Alvarez N, Mckey D, Hossaert-Mckey M, Born C, Mercier L, Benrey B. 2005. Ancient and recent evolutionary history of the bruchid beetle, *Acanthoscelides obtectus* Say, a cosmopolitan pest of beans. Mol Ecol. 14(4):1015–1024. 15773933 10.1111/j.1365-294X.2005.02470.x

[CIT0002] Bocak L, Barton C, Crampton-Platt A, Chesters D, Ahrens D, Vogler AP. 2014. Building the Coleoptera tree-of-life for >8000 species: composition of public DNA data and fit with Linnaean classification. Syst Entomol. 39(1):97–110.

[CIT0003] Crowson RA. 1955. The natural classification of the families of Coleoptera. London (UK): Nathaniel Lloyd.

[CIT0004] Duckett CN. 1997. The scientific method and the predictive value of classification. Chrysomela. 34:3–4.

[CIT0005] Duckett CN, Gillespie JJ, Kjer KM. 2004. Relationships among the subfamilies of Chrysomelidae inferred from small subunit ribosomal DNA and morphology, with special emphasis on the relationship among the flea beetles and the Galerucinae. In: Jolivet PH, Santiago-Baly JA, Schmitt M, editors. New developments in the biology of Chrysomelidae. Boston (MA): Kluwer Academic publishers; p. 3–18.

[CIT0006] Farrell BD, Sequeira AS. 2004. Evolutionary rates in the adaptive radiation of beetles on plants. Evol. 58:1984–2001. 10.1111/j.0014-3820.2004.tb00484.x 15521456

[CIT0007] Haddad S, Mckenna DD. 2016. Phylogeny and evolution of the superfamily Chrysomelidae (Coleoptera: Cucujiformia). Syst Entomol. 41(4):697–716.

[CIT0008] Jin JJ, Yu WB, Yang JB, Song Y, Yi TS, Li DZ. 2018. GetOrganelle: a simple and fast pipeline for de novo assembly of a complete circular chloroplast genome using genome skimming data. bioRxiv. DOI:10.1101/256479.

[CIT0009] Johnson CD. 1970. Biosystematics of the Arizona, California, and Oregon species of the seed beetle genus *Acanthoscelides* Schilsky (Coleoptera: Bruchidae). Univ Calif Publ Entomol. 59:1–116.

[CIT0010] Johnson CD. 1981. Seed beetle host specificity and the systematics of the Leguminosae. In Polhill RM, Raven PH, editors. Advances in legume systematics. London (UK): The Royal Botanic Gardens; p. 995–1027.

[CIT0011] Johnson CD, Southgate BJ, Delobel A. 2004. A revision of the Caryedontini (Coleoptera: Bruchidae: Pachymerinae) of Africa and the Middle East. Mem Am Entomol Soc. 44:1–120.

[CIT0012] Kalyaanamoorthy S, Minh BQ, Wong TKF, Von Haeseler A, Jermiin LS. 2017. ModelFinder: fast model selection for accurate phylogenetic estimates. Nat Methods. 14(6):587–589. 28481363 10.1038/nmeth.4285 PMC5453245

[CIT0013] Katoh K, Standley DM. 2013. MAFFT multiple sequence alignment software version 7: improvements in performance and usability. Mol Biol Evol. 30(4):772–780. 23329690 10.1093/molbev/mst010 PMC3603318

[CIT0014] Li Y, Omar YM, Zhang RZ. 2016. Taxonomic studies on the genus *Caryopemon* (Coleoptera: Chrysomelidae: Bruchinae) of China and Myanmar with some new host plants. Fla Entomol. 99(2):257–263.

[CIT0015] Lingafelter A, Pakaluk J. 1997. Comments on the Bruchinae and Chrysomelidae. Chrysomela. 33:3–4.

[CIT0016] Sayadi A, Immonen E, Tellgren-Roth C, Arnqvist G. 2017. The evolution of dark matter in the mitogenome of seed beetles. Genome Biol Evol. 9(10):2697–2706. 29048527 10.1093/gbe/evx205 PMC5737749

[CIT0017] Schmitt M. 1998. Again, bruchid classification. Chrysomela. 36:3–4.

[CIT0018] Song N, Yin X, Zhao X, Chen J, Yin J. 2018. Reconstruction of mitogenomes by NGS and phylogenetic implications for leaf beetles. Mitochondrial DNA A. 29(7):1041–1050. 10.1080/24701394.2017.1404044 29191077

[CIT0019] Stamatakis A. 2014. RAxML version 8: a tool for phylogenetic analysis and post-analysis of large phylogenies. Bioinformatics. 30(9):1312–1313. 24451623 10.1093/bioinformatics/btu033 PMC3998144

[CIT0020] Tillich M, Lehwark P, Pellizzer T, Ulbricht-Jones ES, Fischer A, Bock R, Greiner S. 2017. GeSeq - versatile and accurate annotation of organelle genomes. Nucleic Acids Res. 45(W1):W6–W11. 28486635 10.1093/nar/gkx391 PMC5570176

[CIT0021] Verma KK. 1998. More on the bruchid controversy. Chrysomela. 35:3.

[CIT0022] Verma KK, Saxena R. 1996. The status of Bruchidae as a family. Chrysomela. 32:3.

[CIT0023] Yao J, Yang H, Dai R. 2017. Characterization of the complete mitochondrial genome of *Acanthoscelides obtectus* (Coleoptera: Chrysomelidae: Bruchinae) with phylogenetic analysis. Genetica. 145(4–5):397–408. 28730527 10.1007/s10709-017-9975-9

